# Hydrocortisone, ascorbic acid, and thiamine (HAT) for sepsis and septic shock: a meta-analysis with sequential trial analysis

**DOI:** 10.1186/s40560-021-00589-x

**Published:** 2021-12-18

**Authors:** Weilan Na, Huili Shen, Yichu Li, Dong Qu

**Affiliations:** 1 Department of Critical Medicine, Children’s Hospital Affiliated to the Capital Institute of Pediatrics, NO.2 Ya Bao Road, Chaoyang District, Beijing, 100020 China; 2 Pediatric Intensive Care Unit, Children’s Hospital of Fudan University, National Children’s Medical Center, Shanghai, 100020 China

**Keywords:** Sepsis, Meta-analysis, Hydrocortisone, Ascorbic acid, Thiamine

## Abstract

**Background:**

Sepsis is a primary global health threat and costs a lot, requiring effective and affordable treatments. We performed this meta-analysis to explore the treatment of hydrocortisone, ascorbic acid, and thiamine (HAT) in sepsis and septic shock.

**Methods:**

We searched Ovid MEDLINE, Embase, and the Cochrane Central Register of Controlled Trials from inception to August 14, 2021. We included randomized controlled trials (RCTs) that evaluated the HAT treatments in sepsis and septic shock. The primary outcome was the change in SOFA score over the 72 h. The second outcomes were the hospital, and 28-/30-day mortality, the duration of vasopressors, PCT clearance, hospital length of stay (LOS), and ICU LOS. We performed a subgroup analysis and a trial sequential analysis (TSA). The Der Simonian–Laird random-effects models were used to report the pooled risk ratios (RR) or mean difference (MD) with confidence intervals (CI).

**Results:**

Nine RCTs, enrolling 1427 patients of sepsis and septic shock treated with HAT (717) or only standard care (710), were included. There was a significant difference between the two groups in the change in SOFA score over the first 72 h (MD 0.65, 95% CI 0.30 to 1.00), the duration of vasopressors (MD − 18.16, 95% CI − 25.65 to − 10.68) and the PCT clearance (MD 14.54, 95% CI 0.64 to 28.43). In addition, there was no significant difference in the hospital mortality (RR 1.07, 95% CI 0.85 to 1.34), the 28-/30-day mortality (RR 0.96, 95% CI 0.80 to 1.15), the hospital LOS (MD 0.78, 95% CI − 0.30 to 1.86), and ICU LOS (MD 0.12, 95% CI − 0.53 to 0.78).

**Conclusions:**

The HAT combination improves the SOFA score in the first 72 h and reduces the duration of vasopressors in patients with sepsis. Given the minor mean difference of the change in SOFA score, the mortality benefit has not been observed.

***Trial registration*:**

PROSPERO, CRD42020203166.

**Supplementary Information:**

The online version contains supplementary material available at 10.1186/s40560-021-00589-x.

## Introduction

Sepsis is a life-threatening organ dysfunction syndrome due to a dysregulated host response to infection [1]. It has been recognized as a primary health threat with high morbidity and mortality, contributing to up to 5.3 million deaths worldwide each year and cost a lot [2]. Given the tremendous financial burden of sepsis, more effective but affordable treatments were required. A retrospective study, conducted by Marik et al. [3], first found that the combination of hydrocortisone, ascorbic acid, and thiamine (HAT) effectively reduced mortality and prevented organ dysfunction for sepsis and septic shock patients.

Hydrocortisone is considered as a typical adjuvant therapy for septic shock, based on the reversal of relative adrenal insufficiency. Low-dose hydrocortisone treatment may rapidly induce hemodynamic stabilization by reducing nitric oxide formation and can regulate the complex immune network in a widely ranging way [4]. Ascorbic acid, well known as Vitamin C, is an important antioxidant and an essential cofactor for biosynthesis and cell metabolization. In patients with sepsis and septic shock, there is a prevalent vitamin C deficiency trend upon admission to intensive care, resulting from increased oxidative stress [5]. Thiamine is referred to vitamin B1, an essential intermediate affecting pyruvate flux to the Krebs cycle. Thiamine deficiency has also been described in septic patients and led to increase lactate production via aerobic metabolism changes [6]. Polypharmacy act synergistically in multiple overlapping ways. This combination's biologic basis is the protective synergistic effect of hydrocortisone and vitamin C that ascorbic acid can restore glucocorticoid receptor function negatively affected by oxide [7]. Septic shock is associated with endothelial barrier dysfunction, which can be synergistically attenuated by hydrocortisone and vitamin C via the reversal of p53 and phosphorylated cofilin downregulation [8]. They also increase tight junctions between endothelial and epithelial cells, which preserves endothelial function and microcirculatory flow. Better yet, both are necessary for the synthesis of catecholamines and increase the sensitivity of vascular vasopressors [9]. In addition, thiamine, with glucocorticoids and vitamin C, can attenuate mitochondrial damage and promote mitochondrial function, which synergistically benefits a lot [10].

The HAT combination is simple, affordable, and theoretically beneficial for septic patients. However, as several RCTs showed conflicting results, the HAT therapy did not appear to reduce the mortality and was not supported for routine use [11–13]. A large retrospective cohort study of US adults with septic shock revealed that the use of HAT therapy increased significantly after Marik et al. [3] proposed the HAT combination, with more than 40% of the study hospitals using it [14]. This early adoption was due to high media attention rather than robust evidence of efficacy, which may carry unintentional risks. When considered in conjunction with recent studies, the combination seems to be a promising treatment, and this meta-analysis aimed to evaluate the effects of hydrocortisone, ascorbic acid, and thiamine given together in sepsis and septic shock.

## Methods

### Data sources and search strategies

The systematic review was performed following the Cochrane Handbook guidelines for Systematic Reviews of Interventions and the PRISMA statement [15, 16]. Trial sequential analysis (TSA) was used to increase the reliability of the meta-analysis and estimate the required information size [17]. The protocol was pre-registered on PROSPERO, ID: CRD42020203166. Ovid MEDLINE, Embase, and the Cochrane Central Register of Controlled Trials (CENTRAL) were searched using the search strategies (Appendix 1) on August 14, 2021. In addition, the reference lists of the included studies and relevant meta-analyses were checked.

### Study selection

Inclusion criteria were as fellow: patients (> 18 years) with sepsis or septic shock; patients receiving HAT treatments in the intervention group; randomized controlled trials. As the HAT combination was first proposed in 2016, the definition of sepsis-3 was accepted. Considering the common use of glucocorticoids in sepsis and septic shock, we did not exclude the use in the control group and all types of glucocorticoids were included. There was no language restriction.

According to the inclusion criteria, two authors independently screened the titles and abstracts and then did full-text reviews of selected studies. Disagreements were resolved by consultation with a third member of the review team.

### Data extraction

Two authors extracted data independently and consensus was reached. The data extracted included the following: authors, publication year, country, study design, number, inclusion and exclusion criteria, demographics, outcome measures and study results, independently.

### Study endpoints

The primary outcome was the change in Sequential Organ Failure Assessment (SOFA) score over 72 h. Secondary outcomes were as follows: the hospital mortality, 28-/30-day mortality, the duration of vasopressors, procalcitonin (PCT) clearance, hospital length of stay (LOS), and ICU LOS.

### Subgroup analysis and sensitivity analysis

For the subgroup analysis, septic shock was assessed as a subgroup. A sensitivity analysis was performed for trials that excluded patients with renal failure at enrollment. For those analyses, the outcome was the change of SOFA score over 72 h.

### Assessment risk of bias

The Cochrane Risk-of-Bias Tool was used to assess the risk of bias in the domains of selection, performance, detection, attrition, and reporting. Two authors completed the assessment independently, and disagreements were resolved by consensus or the third author.

### Statistical analysis

Data were analyzed using Review Manager 5.3 and TSA 0.9.5.10 Beta program. We presented results as forest plots through the risk ratios (RRs) with 95% confidence intervals (CI) for dichotomous data. Forest plots using the mean difference (MD) with 95% CI were performed for continuous data. The heterogeneity was defined via *I*^2^ statistic. An *I*^2^-value > 50% was considered heterogeneity. Random-effects model was used for all pooled analysis. If the value of *P* was less than 0.05, regarded as statistically significant. We also conducted a TSA to control random errors and calculate the required information size (RIS) based on a two-sided $a$ of 0.05, $\beta$ of 80%.

## Results

### Search results and study characteristics

The search retrieved 148 results up to August 14, 2021. After the elimination of duplicates, 113 studies were eligible based on the assessment of the title and abstract.

**Fig. 1:**
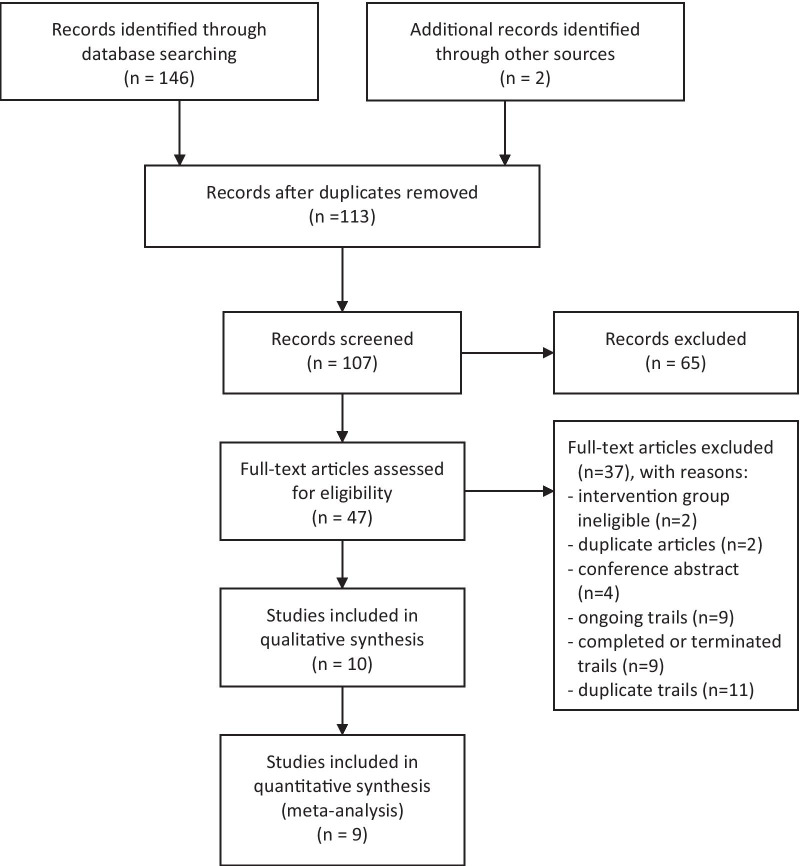
Flow diagram

Then 47 trials were reviewed with the full text; 10 were included in the systematic review and 9 were included in the meta-analysis finally (Fig. 1). The excluded study did not contain the predefined outcomes [18]. One thousand four hundred and twenty seven patients with sepsis and septic shock were included in the meta-analysis (717 in the HAT treatment group and 710 in the control group). The characteristics of each trial were summarized in Table 1. The included studies differed in the application of glucocorticoids. In three studies [11–13], patients in the control group were only treated with the standard care for sepsis and septic shock, including broad-spectrum antibiotics, intravenous fluids, vasopressors, and mechanical ventilation. In another three studies [19–21], intensivists were allowed to order open-label corticosteroid therapy as deemed necessary. In three other studies [22–24], patients in the control group were routinely given low doses of glucocorticoids. In addition, the severity of sepsis was varied. Four trials focused on patients with sepsis including those with septic shock [11, 12, 19, 21] and the others only focused on patients with septic shock [13, 20, 22–24].

**Table 1:** Characteristics of included studies

References	Country	Inclusion criteria	Exclusion criteria	Group	Samples	Age	Sex (male/female)	Intervention protocol	Outcomes	Authors’ conclusions
Moskowitz [13]	United States	1. Age: ≥ 18 years 2. Septic shock	1. Allergic to study drug components 2. Had a clinical indication for the study drugs 3. Had kidney stones last year 4. Had G6PD deficiency or hemochromatosis 5. Receiving RRT	HAT	101	68.9	57/44	Ascorbic acid (1.5 g) and thiamine (100 mg): mixed in 100 mL of normal saline and administered intravenously every 6 h for 4 days or discharge from ICU Hydrocortisone: 50 mg/1 ml IV push every 6 h for 4 days or discharge from ICU	Primary outcome: 1. Change in SOFA score between enrollment and 72 h Secondary outcomes: 1. All-cause mortality over 30 day 2. Kidney failure 3. Ventilator-free days 4. Shock-free days 5. Incidence of delirium 6. ICU-free days 7. All-cause mortality to ICU discharge 8. All-cause mortality to hospital discharge 9. Survivors discharged home	The HAT therapy was not supported routine use for patients with septic shock
				Control	99	67.7	54/45	0.9% sodium chloride: the same frequency and volume as above		
Wani [11]	India	Sepsis and septic shock with a serum lactate level of > 2 mmol/l	1. Age: < 18 years 2. Pregnancy	HAT	50	65	28/22	Vitamin C: 1.5 g/100 ml IV piggyback every 6 h for 4 days or discharge from the hospital Thiamine: 200 mg/50 ml IV piggyback every 12 h for 4 days or discharge from the hospital Hydrocortisone: 50 mg every 6 h for 7 days or until ICU discharge followed by a taper over 3 days	Primary outcomes: 1. In-hospital mortality Secondary outcomes: 1. 30-day mortality 2. Duration of hospital stay 3. Duration of vasopressor therapy 4. Lactate clearance 5. Change in serum lactate 6. The SOFA score over the first 4 days	Addition of HAT therapy into standard care of sepsis does not improve in-hospital or 30-day mortality. However, lower vasopressor requirement and faster lactate clearance is observed with treatment
				Control	50	70	31/19	–		
Iglesias [19]	United States	1. age: ≥ 18 years 2. Sepsis or septic shock ≦ 24 h from admission	1. Pregnancy 2. DNR/DNI 3. Had a terminal end stage disease or G-6PD deficiency 4. Required surgery 5. HIV and a CD4 < 50 mm^2^ 6. Transferred from another hospital	HAT	68	70	32/36	Ascorbic acid: 1.5 g/100 ml IV piggyback every 6 h for 4 days or discharge from ICU Thiamine: 200 mg/50 ml IV piggyback every 12 h for 4 days or discharge from ICU Hydrocortisone: 50 mg IV push every 6 h for 4 days or discharge from ICU	Primary outcome: 1. Duration of vasopressors 2. Change in SOFA score at 72 h Secondary outcomes: 1. ICU mortality 2. Hospital mortality 3. Hospital LOS 4. ICU LOS 5. PCT clearance 6. Ventilator-free days 7. AKI	The HAT therapy significantly reduced the time to resolution of shock
				Control	69	67	27/42	Sodium Chloride 0.9%: the same frequency and volume as above		
Chang 2020 [12]	China	A primary diagnosis of sepsis or septic shock and PCT level ≥ 2 ng/ml	1. Age: < 18 years 2. Pregnancy 3. Patients with limitations of care	HAT	40	59.5	22/18	Vitamin C: 1.5 g every 6 h for 4 days or until ICU discharge Thiamine: 200 mg every 12 h for 4 days or until ICU discharge Hydrocortisone: 50 mg every 6 h for 7 days or until ICU discharge	Primary outcomes: 1. 28-days mortality Secondary outcomes: 1. ICU LOS 2. Duration of vasopressors 3. New AKI after entering ICU 4. Change in SOFA score at 72 h 5. PCT clearance 6. Duration mechanical ventilation 7. Lactate clearance	The HAT therapy did not appear to reduce the 28-day mortality compared with placebo in patients with sepsis or septic shock
				Control	40	63.7	21/19	Normal saline: 500 ml every day for 4 days, then 200 ml every day for 3 days		
Fujii 2020 [22]	Australia; Japan	A primary diagnosis of septic shock at enrollment	1. Age < 18 years 2. Pregnancy 3. DNR 4. Imminent death 5. Diagnosis of septic shock longer than 24 h ago 6. Disease as indication or contraindication for any of the study drugs	HAT	107	61.9	68/39	Intravenous vitamin C (1.5 g every 6 h), hydrocortisone (50 mg every 6 h) Thiamine (200 mg every 12 h)	Primary outcome: 1. Time alive and free of vasopressors Secondary outcomes: 1. 28-days mortality 2. 90-days mortality 3. ICU mortality 4. Hospital mortality 5. 28-days cumulative vasopressor-free days 6. 28-days RRT-free days 7. Change in SOFA score at day 3 8. 28-days ICU free days	In patients with septic shock, HAT treatment, did not significantly improve the duration of time alive and free of vasopressor administration over 7 days HAT does not lead to a more rapid resolution of septic shock
				Control	104	61.6	65/39	IV hydrocortisone (50 mg every 6 h)		
Hussein 2021 [23]	Egypt	1. Adult 2. Septic shock	1. Pregnancy 2. Lactation 3. Refusal of attending 4. Had disease as indication or contraindication to any of the study drugs 5. Immunosuppressive medications 6. Oncology patients 7. DNR/DNI	HAT	47	65.81	25/22	Hydrocortisone 50 mg/6-h IV for 7 days or ICU discharge vitamin C 1.5 g/6-h IV for 4 days or till ICU discharge Thiamine 200 mg/12-h IV for 4 days or till ICU discharge	Primary outcome: 1. 28-day in-hospital mortality 2. ICU mortality Secondary outcomes: 1. Duration on vasopressors 2. Weaning from mechanical ventilation 3. Improvement of organ function (Scr, AST, ALT) 4. Improvement of septic markers (TLC, CRP, lactate, PCT)	The HAT therapy showed a non-significant reduction in 28-day mortality and SOFA score but significantly lower shock time and duration on vasopressor use
				Control	47	61.6	26/21	Hydrocortisone 50 mg/6-h IV for 7 days or till ICU discharge		
Mohamed [20]	India	Adult non-pregnant patients with septic shock and within 6 h of initiation of inotropic support	Patients with burns, limitations of care due to terminal illness or acute liver failure	HAT	45	58.69	31/14	intravenous combination of vitamin C: 1.5 g q6h thiamine: 200 mg q12h Hydrocortisone: 50 mg q6h first doses of the drugs administered within 6 h of onset of septic shock admission	Primary outcome: 1. All-cause mortality during inpatient stay Secondary outcomes: 1. Time to shock reversal 2. Change in SOFA score over 72 h 3. Need for mechanical ventilation 4. Incidence of new onset of AKI 5. ICU and hospital LOS	HAT protocol did not reduce hospital mortality in patients with septic shock HAT group has higher incidence of culture positivity for Klebsiella and Candida; significant reduction of hock reversal time, PCT level on day 3, PCT clearance at 72 h and ICU length of stay
				Control	45	59.37	32/11	standard of care for septic shock The use of hydrocortisone and vitamin supplements in the control group was at the treating physician’s discretion		
Reddy 2020 [24]	India	Septic shock Age ≥ 18 years	Pregnancy have new onset acute coronary syndrome	H	7	55.4	3/4	Hydrocortisone:200 mg over 24 h infusion	Primary outcome: 1. Time to shock reversal Secondary outcomes: 1. time to vasopressor reduction (minutes) from SOFA(h) 4–3	H group has non-significantly longer shock reversal time than other groups No significant difference in time to initiation of metabolic resuscitation among groups Did not include the length of stay, ventilator-free days, and mortality in outcomes
				HA	7	56.5	3/4	Hydrocortisone:200 mg over 24 h infusion ascorbic acid 1.5 g IV q6 hour		
				HAT	7	53.8	4/3	Hydrocortisone:200 mg over 24 h infusion ascorbic acid 1.5 g IV q6 hour thiamine:200 mg IV q12 hours		
Sevransky [21]	US	Age ≥ 18y Acute respiratory and/or cardiovascular dysfunction caused by sepsis ICU admission	Age < 18y Weight < 40 kg Organ dysfunction no longer present Cardiovascular/respiratory organ failure caused by other disease DNR, DNI hospitalization > 30 days indication or contraindication of any study drug	HAT	252	62	139/113	IV vitamin C (1.5 g), thiamine hydrochloride (100 mg), and hydrocortisone sodium succinate (50 mg) within 4 h, then q6 hours thereafter up to 96 h, death, or discharge from the ICU	Primary outcome: 1. The number of consecutive VVFDs in the first 30 days Secondary outcomes 1. mortality within 30 days of randomization 2. ICU mortality 3. ICU and hospital LOS 4. ICU delirium- and coma-free days 5. Kidney replacement therapy-free days at day 30 6. Change in SOFA score (DAY4)	HAT compared with placebo, did not significantly increase ventilator- and vasopressor free days within 30 days
				Control	249	61	134 /115	Matching placebos within 4 h, and then q6 hours thereafter up to 96 h, death, or discharge from the ICU		

*RRT* renal replacement therapy; *DNR* do not resuscitate; *DNI* do not intubate; *CRF* chronic renal failure; *SOFA* Sequential Organ Failure Assessment; *LOS* length of stay; *TLC* total leukocytic count; *CRP* C-reactive protein; *PCT* procalcitonin; *AKI* acute kidney injury; *RRT* renal replacement therapy; *VVFDs* ventilator and vasopressor free days

### Outcomes

**Fig. 2:**
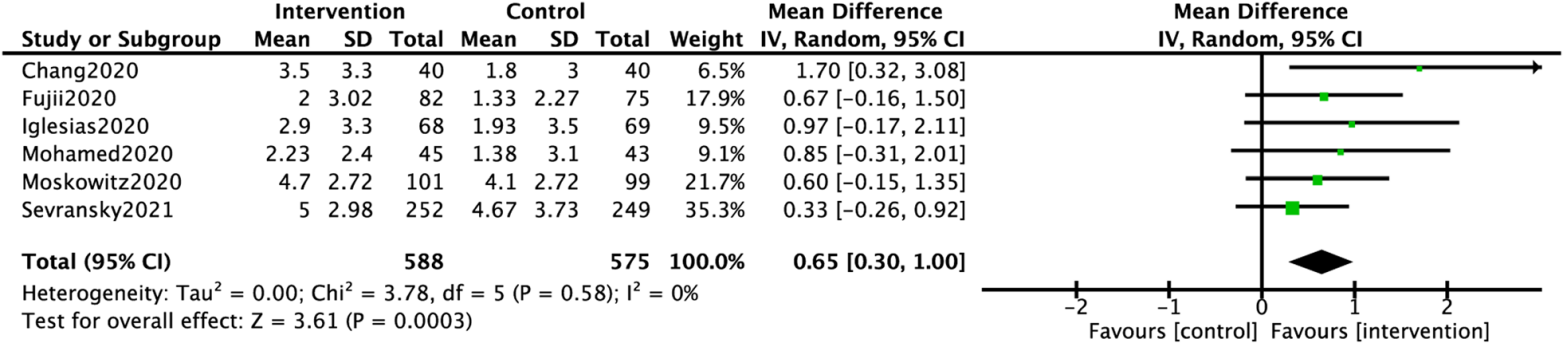
Forest plot of the primary outcome. *Legends* Forest plot of the change in SOFA score over the first 72 h in the comparison between HAT treatment and control in sepsis and septic shock

The forest plot of the primary outcomes was shown in Fig. 2. The change in SOFA score over 72 h was reported in six studies (588 in the HAT group and 575 in the control group). A significant reduction in SOFA score was revealed, with the use of HAT, and there was no significant heterogeneity (MD 0.65, 95% CI 0.30 to 1.00, *P* = 0.0003; *I*^2^ = 0%, *P*_*H*_ = 0.58).

**Fig. 3:**
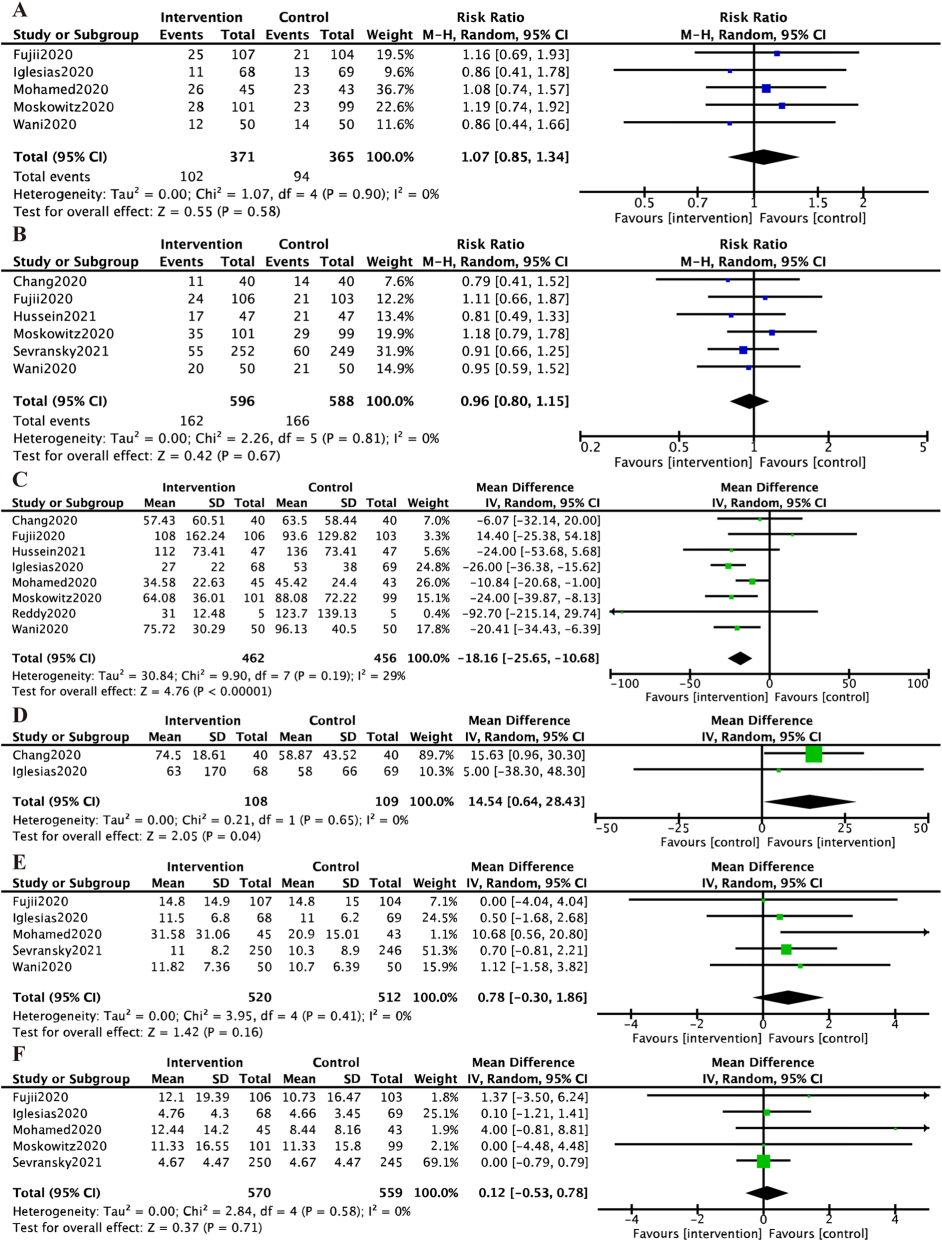
Forest plots of the second outcomes. *Legends* Forest plots of the hospital mortality (**a**), 28-/30-day mortality (**b**), duration of vasopressors (hours) (**c**), procalcitonin clearance (**d**), hospital LOS (**e**), and ICU LOS (**f**) in the comparison between HAT treatment and control in sepsis and septic shock

For the secondary outcomes, the pooled RR of hospital mortality and 28-/30-day mortality did not reach the statistical significance (RR 1.07, 95% CI 0.85 to 1.34 and RR 0.96, 95% CI 0.80 to 1.15, respectively) (Fig. 3a and b). The pooled results of the duration of vasopressors revealed a significant reduction in the HAT treatment group, with no heterogeneity (MD − 18.16, 95% CI − 25.65 to − 10.68, *P* < 0.01; *I*^2^ = 29%, *P*_*H*_ = 0.65; Fig. 3c). For the PCT clearance, there was statistical significance between two groups (MD 14.54, 95% CI 0.64 to 28.43; Fig. 3d). In addition, there were no significant differences in the hospital and ICU LOS between the two groups with pooled MD of 0.78 (95% CI − 0.30 to 1.86) and 0.12 (95% CI − 0.53 to 0.78), respectively (Fig. 3e and f).

**Fig. 4:**
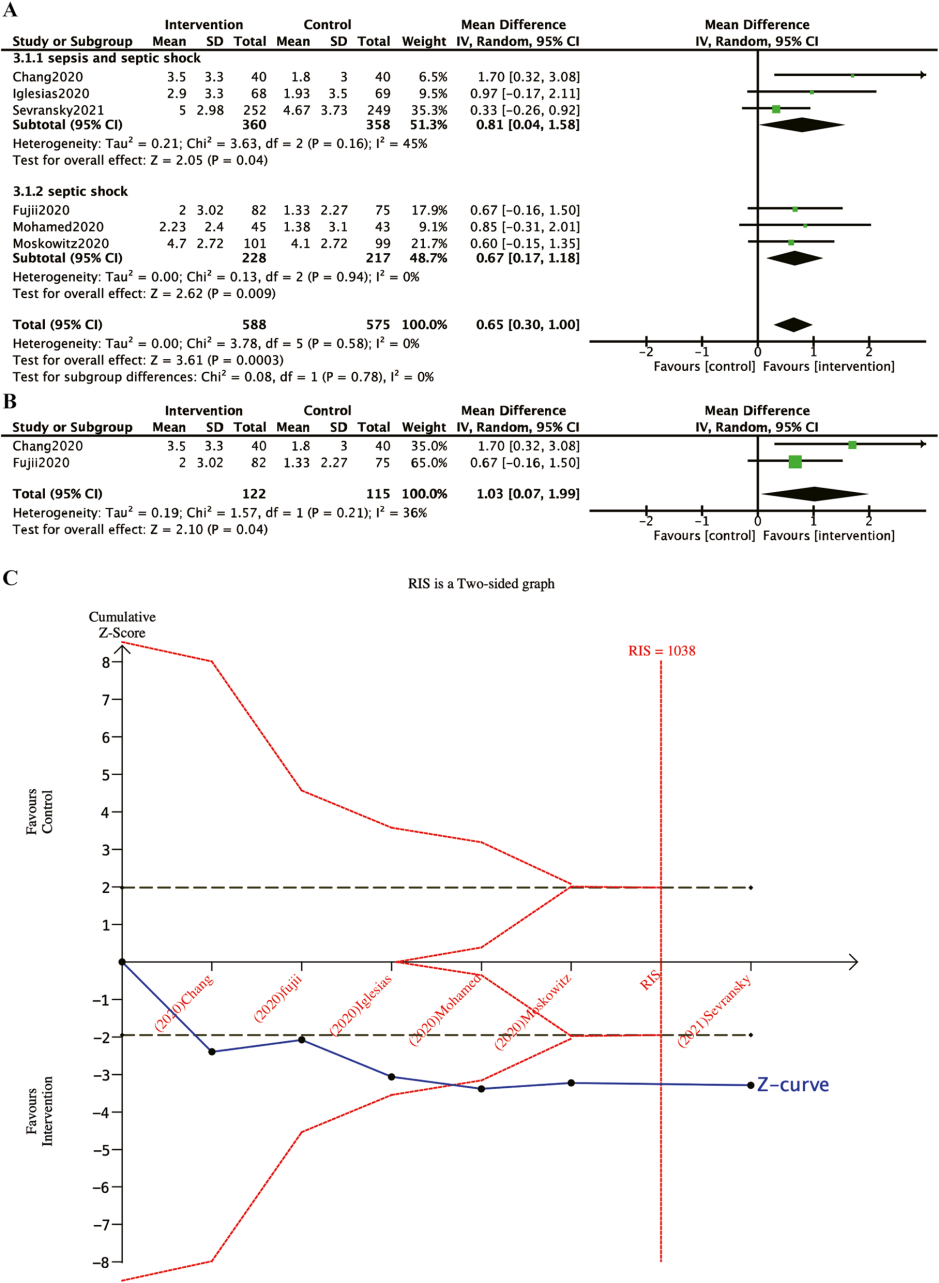
Subgroup analysis, sensitivity analysis and TSA of the primary outcome. Legends: **a** Forest plots of the subgroup analysis for the change in SOFA score over the first 72 h. Septic shock was assessed as a subgroup. **b** Forest plots of the sensitivity analysis for the change in SOFA score over the first 72 h. Trials that excluded patients with renal failure at enrollment was assessed. **c** Trial sequential analysis for the change in SOFA score over the first 72 h. The blue cumulative z curve crossed the conventional monitoring boundary and the red trial sequential boundary for benefit (the pooled effect, 0.56; 95% CI 0.23–0.89; *I*^2^ = 0%). The required information size (RIS) was 1038 (a two-sided a of 0.05, *β* of 80%)

For the subgroup analysis of septic shock, the result was presented in Fig. 4a. Four in seven studies were included and the HAT treatment showed a significant improvement in the SOFA score over 72 h (MD 0.67, 95% CI 0.17 to 1.18). For the sensitivity analysis, only two trials excluded the patients with renal failure at enrollment and there was also statistically significant (MD 1.03, 95% CI 0.07 to 1.99; Fig. 4b).

### Trial sequential analysis results

TSA showed the adjusted pooled effect of the change in SOFA score over 72 h was 0.56 (95% CI 0.23 to 0.89) (Fig. 4c). The red cumulative *z* curve crossed the blue trial sequential boundary and the conventional boundary, indicating that the result was stable and statistically significant. In addition, the RIS of 1038 patients had been accrued, which indicated a sufficient number of studies.

### Publication bias and risk of bias

**Fig. 5:**
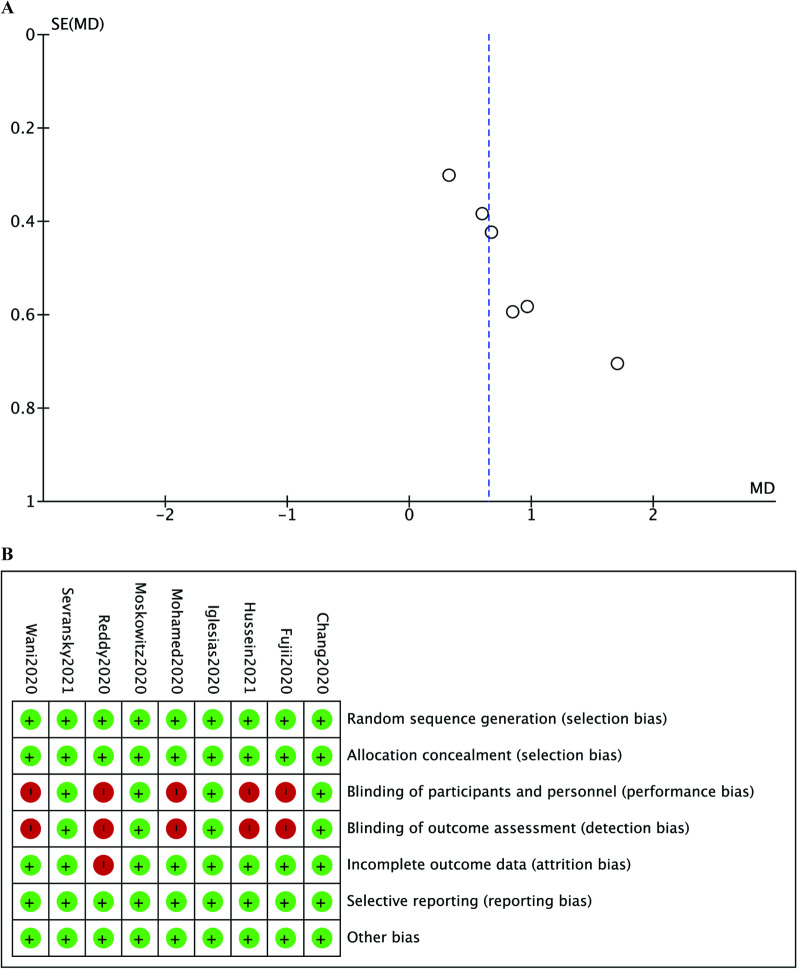
Funnel plot and risk of bias summary. Legends: **a** Funnel plot assessing publication bias. The dots represent individual studies. **b** risk of bias summary for the included studies

The presence of publication bias for the primary outcome was tested and the funnel plot did not show the existence of publication bias via a visual inspection (Fig. 5a). For the risk of bias, the lack of blinding led to the performance bias and detection bias rated the highest (high risk of biases in 5/10 trials) (Fig. 5b).

## Discussion

In this systematic review, the combination of hydrocortisone, ascorbic acid, and thiamine led to the reduction of SOFA score over 72 h, the duration of vasopressors and the improvement of PCT clearance. However, the HAT combination did not show benefit in the mortality, the duration of mechanical ventilation, and hospital or ICU LOS.

According to the Sepsis-3 clinical criteria, the diagnosis of sepsis has emphasized organ dysfunction which was represented by the increase of two points or more SOFA score. In addition, a change in the SOFA score was accepted by the European Medicines Agency as a surrogate marker of efficacy in exploratory trials of novel therapeutic agents in sepsis [25]. Moreover, organ dysfunction was associated with about 10% increase in mortality [26]. Therefore, we selected the change of SOFA score over the 72 h, rather than hospital mortality which was chosen in the PROSPERO registry, as the primary outcome. In this meta-analysis, there was a significant effect of the intervention on the change of SOFA score, but this did not be translated into a mortality benefit. First, hospital mortality is all-cause mortality and is influenced by many factors, such as comorbidities. Most included studies did not exclude the patients with a terminal end-stage disease or with imminent death, which may underestimate the therapeutic effect. In addition, the pooled effect (MD 0.65, 95% CI 0.30 to 1.00) was so minor and did not achieve the minimal clinically important difference, which was set as a 2-point difference [1, 13]. However, caution should be paid to the interpretation of the statistical results. It is valid only if the SOFA change is clinically relevant. The SOFA score focused on the early recovery of organ function and was assessed only if the patients remained in the ICU on the third day. Considering of the potential endpoints, such as death or recovery leading to early discharge from ICU, which increased the bias of competing risk [22]. Although the pooled effect of both meta-analysis and TSA supported the reliability and stability, the mean difference was small and we should still be cautious to evaluate the effect of HAT combination on the organ function.

Numerous retrospective studies showed a conflicting result on the mortality benefits, but the HAT combination did not provide significant survival benefits in this meta-analysis. In the study of Wald et al. [27], the HAT combination was found to be associated with lower mortality in pediatric septic shock, and the improvement seemed to be primarily associated with reduced early deaths. In addition, Marik et al. [3] found that the early use of the HAT combination appeared to significantly affect patients' hospital mortality with sepsis and septic shock. According to clinical pharmacologic knowledge and pathophysiological mechanisms, we speculated that the early use of the HAT combination may make sense for patients at different sepsis stages. Noteworthy, the HYVCTTSSS trial, conducted by Chang et al. [12], showed that the HAT group got a better therapeutic effect than the control group in the subgroup, where patients were diagnosed with sepsis within 48 h, reflected mainly in the improvement of mortality. Hence, there is reason to believe that early treatment can lead to higher survival rates.

Theoretically, glucocorticoids and vitamin C have the ability to synergistically increase the sensitivity of vasopressors, which was also reflected in the results of this meta-analysis. In the HAT group, the duration of vasopressors was significantly reduced. Early liberation from vasopressor therapy means early recovery from septic shock. Although it could provide a more stable hemodynamic basis for subsequent treatment, the infectious source control remains the key for the mortality. The prognostic value of serum PCT in septic patients has been widely investigated and PCT non-clearance are strongly associated with all-cause mortality [28, 29]. Although there was statistically significant, caution should be exercised when interpreting the unstable results. In the ViCTOR Trial [20], the hospital LOS was significantly higher in the HAT group. However, after adjusting for outliers, the average LOS between the study groups did not significantly differ, which was consistent with the pooled effect of this meta-analysis.

Two meta-analyses regarding the effects of HAT therapy were published recently. Both Zayed et al. [30] and Somagutta et al. [31] concluded that HAT therapy significantly improved the SOFA score but appeared not to have significant benefits in the mortality, which was consistent with the results of this meta-analysis with a larger sample size. However, in our meta-analysis, instead of focusing on mortality, we set the change in SOFA score as the primary outcome. This is the first meta-analysis that placed importance on the effects of HAT therapy on organ function. We also discussed the pathophysiologic basis and the synergistic effects for these three drugs. Moreover, we conducted TSA, subgroup analysis and sensitivity analysis to enhance methodological quality. The VICTAS trial was a large multicenter RCT, published by Sevransky et al. [21] on JAMA, which enrolled 501 patients from 43 hospital in America. With inclusion of the VICTAS trial, the results of TSA showed that the RIS was reached, and the statistical results were significant and stable. In addition, this meta-analysis is the largest at present, with nine RCTs included.

Several limitations should also be considered. First, five trials of all included were lack of blinding, which is the association with underestimation of adverse effects. Moreover, this review did not focus on side effects. The HYVCTTSSS trial was terminated at interim analysis due to the significant incidence of hypernatremia in the HAT group [12]. Intravenous high dose of vitamin C in patients with renal failure was likely to increase oxalate, which eventually metabolized through kidneys, put their kidneys under stress [32]. Nevertheless, those could be managed in ICU. Finally, this review did not conduct more subgroup analysis. Optimal dosing time, dosage, and the administration of glucocorticoid should be considered to guide clinical practice.

## Conclusions

The HAT combination improves the SOFA score in the first 72 h and reduces the duration of vasopressors in patients with sepsis. Given the minor mean difference of the change in the SOFA score, the mortality benefit has not been observed.

## Supplementary Information

**Additional file 1: Appendix 1**. Search strategies

## Data Availability

The data and materials for this meta-analysis are included in the list of references.

## Abbreviations

HAT: Hydrocortisone, ascorbic acid, and thiamine
RCTs: Randomized controlled trials
LOS: Length of stay
TSA: Trial sequential analysis
RR: Risk ratios
MD: Mean difference
CI: Confidence intervals
CENTRAL: Cochrane Central Register of Controlled Trials
SOFA: Sequential Organ Failure Assessment
PCT: Procalcitonin
RIS: Required information size
