# Supplementary material for: Hydrocortisone, ascorbic acid, and thiamine (HAT) for sepsis and septic shock: a meta-analysis with sequential trial analysis
Source: J Intensive Care. 2021 Dec 18;9:75. doi: 10.1186/s40560-021-00589-x (PMC8684090; doi:10.1186/s40560-021-00589-x)
Supplement: Supplementary file 1 — Additional file 1: Appendix 1. Search strategies [file 40560_2021_589_MOESM1_ESM.docx]

**Appendix 1. search strategies**

**Cochrane**

#1 MeSH descriptor Sepsis explode all trees

#2 MeSH descriptor Shock, Septic explode all trees

#3 MeSH descriptor Systemic Inflammatory Response Syndrome explode all trees

#4 (sepsis or septic* or blood?stream infection* or (shock adj3 (endotoxic or toxic)))

#5 #1 or #2 or #3 or #4

#6 ascorb* or (Vit* near/6 C) or antioxidant*

#7 MeSH descriptor Adrenal Cortex Hormones explode all trees

#8 MeSH descriptor Steroids explode all trees

#9 steroid* or corticosteroid* or glucocorticoid* or hydrocortison*

#10 #7 or #8 or #9

#11 thiamin* or aneurin* or (Vit* near/6 B1)

#12 #6 and #10 and #11

#13 #5 and #12

**Medline-Ovid**

1 exp Sepsis/ or exp Shock Septic/ or exp Septicemia/ or exp Systemic Inflammatory Response Syndrome

2 (sepsis or septic* or blood?stream infection* or (shock adj3 (endotoxic or toxic))).mp.

3 1 or 2

4 (ascorb* or (Vit* adj6 C) or antioxidant*).mp.

5 exp Adrenal Cortex Hormones/ or exp Steroids

6 (steroid* or corticosteroid* or glucocorticoid* or hydrocortison*).mp.

7 5 or 6

8 (thiamin* or aneurin* or (Vit* adj6 B1)).mp.

9 4 and 7 and 8

10 "randomized controlled trial".pt.

11 (random$ or placebo$ or single blind$ or double blind$ or triple blind$).ti,ab.

12 (retraction of publication or retracted publication).pt.

13 or/11-13

14 (animals not humans).sh.

15 ((comment or editorial or meta-analysis or practice-guideline or review or letter or journal correspondence) not "randomized controlled trial").pt.

16 (random sampl$ or random digit$ or random effect$ or random survey or random regression).ti,ab. not "randomized controlled trial".pt.

17 14 not (15 or 16 or 17)

18 3 and 9 and 17

**Embase**

#1 'sepsis'/exp OR 'shock septic'/exp OR 'septicemia'/exp OR 'systemic inflammatory response syndrome'/exp

#2 sepsis:ti,ab OR septic*:ti,ab OR ‘blood$stream infection*’:ti,ab OR ((shock NEAR/3 (endotoxic OR toxic)):ti,ab)

#3 #1 or #2

#4 ascorb*:ti,ab or ((Vit* NEAR/6 C):ti,ab) or antioxidant*:ti,ab

#5 ‘Adrenal Cortex Hormones’/exp or ‘Steroids’/exp

#6 steroid*:ti,ab or corticosteroid*:ti,ab or glucocorticoid*:ti,ab or hydrocortison*:ti,ab

#7 #5 or #6

#8 thiamin*:ti,ab or aneurin*:ti,ab OR ((Vit* NEAR/6 B1):ti,ab)

#9 #4 and #7 and #8

#10 'randomized controlled trial'/exp or 'randomization'/exp or 'controlled study'/exp or 'double blind procedure'/exp or 'single blind procedure'/exp

#11 ((singl* OR doubl* OR trebl* OR tripl*) NEAR/5 (blind* OR mask*)):ti,ab

#12 random*:ti,ab OR 'cross* over*':ti,ab OR factorial*:ti,ab OR placebo*:ti,ab OR volunteer*:ti,ab

#13 human*:ti,ab

#14 animal*:ti,ab OR nonhuman*:ti,ab

#15 #13 AND #14

#16 #14 NOT #15

#17 #10 OR #11 OR #12

#18 #17 NOT #16

#19 #3 AND #9 AND #18
